# Effect of renal denervation on urine angiotensinogen excretion in prenatally programmed rats

**DOI:** 10.14814/phy2.13482

**Published:** 2017-10-19

**Authors:** Asifhusen Mansuri, Susan K. Legan, Jyoti Jain, Issa Alhamoud, Jyothsna Gattineni, Michel Baum

**Affiliations:** ^1^ Department of Pediatrics University of Texas Southwestern Medical Center at Dallas Dallas Texas; ^2^ Department of Internal Medicine University of Texas Southwestern Medical Center at Dallas Dallas Texas

**Keywords:** Angiotensinogen, prenatal programming, renal denervation

## Abstract

Prenatal programming results in an increase in blood pressure in adult offspring. We have shown that compared to control adult offspring whose mothers were fed a 20% protein diet, programmed adults whose mothers were fed a 6% protein diet during the last half of pregnancy have an increase in renal sympathetic nerve activity and urinary angiotensinogen/creatinine levels. We hypothesized that the increase in urinary angiotensinogen was mediated by renal sympathetic nerve activity in programmed rats. In this study performed in 3 month old rats, renal denervation resulted in normalization of blood pressure in the 6% programmed group (150 ± 3 Hg in 6% sham vs. 121 ± 4 Hg in 6% denervated, *P* < 0.001), and a reduction in blood pressure in the 20% group (126 ± 2 Hg 20% sham vs. 113 ± 4 Hg 20% denervated (*P* < 0.05). We confirm that the intrarenal renin–angiotensin system assessed by urinary angiotensinogen/creatinine is upregulated in offspring of rats fed a 6% protein diet rats compared to 20% controls. To determine if sympathetic nerve activity was mediating the increase in urinary angiotensinogen in programmed rats, we compared denervated to sham‐operated control and programmed rats. Renal denervation had no effect on urinary angiotensinogen/creatinine ratio in the 20% group and no effect on the increased urinary angiotensinogen/creatinine ratio found in programmed rats. This study demonstrates that the increase in urinary angiotensinogen in programmed rats is not mediated by renal sympathetic nerve activity.

## Introduction

David Barker and his colleagues demonstrated that infants born at a low birth weight are at increased risk for development of hypertension as adults (Barker et al. 1990). He followed up on this observation by showing that the effect of birth weight on blood pressure increased as subject's age. The effect of birth weight on blood pressure was small in children, however, the blood pressure was quite elevated in the elderly who were born small for gestational age (Law et al. 1993). While the association of low birth weight and adult hypertension has been replicated in numerous populations, the cause for the hypertension in small for gestational age infants has been enigmatic.

To understand the pathogenesis of how small for gestational age neonates develop hypertension in later life, studies have utilized animal models that replicate fetal insults that result in low birthweight offspring. Prenatal programming with maternal dietary protein and caloric restriction, uteroplacental insufficiency, and prenatal administration of glucocorticoids in rats result in offspring that develop hypertension as adults (Merlet‐Benichou et al. 1994; Langley‐Evans et al. 1996, 1999a,b; Ortiz et al. 2001; Alexander 2003). While the etiology of the hypertension may be multifactorial, there is considerable evidence that renal sympathetic nerve activity plays a major role in the pathogenesis of the hypertension in programmed rats. Renal denervation has been shown to normalize the blood pressure in programmed adult male rats whose mothers had surgically induced reduced uterine perfusion and in male rats whose mothers were administered prenatal dexamethasone compared to sham‐operated controls (Alexander et al. 2005; Dagan et al. 2008).

In addition to renal sympathetic nerve activity being a factor in the pathogenesis of hypertension, recent studies have pointed to the role of the intrarenal renin–angiotensin system playing a role in the hypertension in programmed rats (Mansuri et al. 2015; Murano et al. 2015). We have recently demonstrated that prenatal programming due to maternal dietary protein deprivation had no significant effect on the systemic renin–angiotensin system, but compared to control rats, programmed rats had a significant increase in urinary angiotensinogen/creatinine, a marker of the intrarenal renin–angiotensin system (Mansuri et al. 2015). Angiotensin converting enzyme inhibition reduced blood pressure and resulted in a marked decrease in urinary angiotensinogen/creatinine levels in programmed rats (Mansuri et al. 2015). An increase in urinary angiotensinogen has been found in rats programmed by maternal uteroplacental insufficiency as well (Murano et al. 2015). Since renal nerves have been shown to regulate the intrarenal renin–angiotensin system and thus urinary angiotensinogen excretion (Nakamura and Johns 1995; Pontes et al. 2015), we aimed to determine if renal nerves mediated the increase in the intrarenal renin–angiotensin system seen in prenatal programming by performing renal denervation in programmed and control rats.

## Methods

### Animals

Pregnant Sprague–Dawley rats were fed either a standard 20% protein or a 6% low protein diet from day 12 of pregnancy until they deliver as we and others have described previously (Vehaskari et al. 2001, 2004; Habib et al. 2011; Mizuno et al. 2013; Mansuri et al. 2015, 2017). We have shown that this protocol does not affect the number of pups (11.4 ± 0.6 pups in the 20% vs. 13.0 ± 0.5 pups in the 6% group, *P *= ns) (Habib et al. 2011). All rats were fed a standard 20% protein diet after birth. The rats were weaned at 3 weeks of age. No more than three rats per litter per group were studied and only male rats were studied to reduce variability and males are more severely affected by prenatal programming than females (Alexander 2003; Ortiz et al. 2003; Woods et al. 2005; Moritz et al. 2009). A total of six litters in the 20% and 6 litters in the 6% group were used. The IACUC of the University of Texas Southwestern Medical Center approved these studies.

### Renal denervation

Renal denervation was performed using the same technique we have published previously (Quan and Baum 2001; Dagan et al. 2008). Briefly, 2‐month‐old rats were anesthetized using ketamine (5 mg/100 g body weight) and xylazine (0.5 mg/100 g body weight). The rats were placed on a heated table to maintain their body temperature at 37°C. A midline incision was made and the renal arteries were exposed. A surgical microscope (Zeiss, Germany) was used to view the renal arteries which were isolated and the adventitia was removed. The entire circumference of each renal artery was coated with 10% phenol as described previously (Quan and Baum 2001; Dagan et al. 2008). The sham‐operated rats had a vertical incision and the kidneys were visualized but not touched. The muscle layer was closed using 3‐0 silk sutures and the rat's skin was closed with staples. The rats were placed in separate cages and allowed to wake. They were given chow allowed to drink after surgery. Pain was controlled with 5 mcg/100 g body weight buprenorphine given immediately after the procedure and a second dose was administered 24 h after surgery. At 3 months of age the rat kidneys were harvested, immediately frozen in liquid nitrogen and stored at −80°C. Renal norepinephrine content was assessed to confirm renal denervation using a norepinephrine ELISA Assay, (ALPCO, Salem, NH). The norepinephrine content of sham and denervated rats is shown in Table 1.

**Table 1:** Effect of prenatal programming and renal denervation on renal norepinephrine content, urinary protein, and albumin excretion

	Kidney Norepinephrine content (ng/gm)	24 h urine protein (mg/24 h)	Protein/creatinine	24 h urine albumin (mg/24 h)	Albumin/creatinine
20% Sham (*n* = 14)	245 ± 25^a^	11.9 ± 1.4	1.9 ± 0.2	4.3 ± 0.9	0.7 ± 0.1
6% Sham (*n* = 15)	274 ± 32^a^	20.9 ± 1.5	3.3 ± 0.2	12.0 ± 2.5^b^	1.9 ± 0.4^b^
20% Denervation (*n* = 12)	34 ± 8	13.2 ± 4.2	2.0 ± 0.6	3.3 ± 1.1	0.5 ± 0.1
6% Denervation (*n* = 13)	40 ± 6	24.0 ± 5.0	4.1 ± 0.8^b^	8.0 ± 2.4	1.3 ± 0.3^c^

a *P* < 0.001 versus denervation.

b *P* < 0.05 versus 20% sham and 20% denervation.

c *P* < 0.05 versus 20% denervation by Student's t‐test.

### Urine collection and measurement of protein, albumin, creatinine, and angiotensinogen

Approximately 2 weeks after surgery, rats were placed in metabolic cages and given ad lib access to food and water. During the first 48 h the rats were acclimated to their environment. A 24 h urine collection was then commenced in a tube containing 300 nmol enalaprilat, 125 *μ*mol EDTA, 10 mg sodium azide, and 50 *μ*mol pepstatin similar to that used described by our laboratory and others to prevent angiotensinogen degradation (Kobori et al. 2002, 2003; Dagan et al. 2010; Mansuri et al. 2015). Urine was also assayed for protein using the Bradford assay (Bio‐Rad Laboratories; Hercules, California), albumin (Nephrat rat urinary albumin enzyme immunoassay kit; Exocell; Philadelphia, Pennsylvania), creatinine (capillary electrophoresis), and angiotensinogen (ELISA Rat Total Angiotensinogen Assay Kit, Immuno‐Biological Laboratories Co, Minneapolis, MN).

### Blood pressure

The week after being placed in metabolic cages, rats were restrained in a Lucite tube daily for 4 days and a cuff was inflated around the rat's tail several times to acclimate the animal to the procedure. On the fifth day at least five blood pressure readings were measured. The mean of all readings was used as the blood pressure for the rat. We have utilized this procedure in prior studies (Mizuno et al. 2013, 2014; Mansuri et al. 2015). The investigator who measured the blood pressures did not know the origin of the rat. A CODA Blood Pressure Non‐Invasive Pressure Analyzer (Kent Scientific Corporation, Torrington, CT) was utilized to measure the blood pressure. The volume pressure recording has been shown to correlate with measurements made using telemetry (Feng et al. 2008). While our protocol minimizes stress taking blood pressure, it is not eliminated. Telemetry eliminates stress, but results in comparable blood pressures in programmed and control rats (O'Regan et al. 2008; Mizuno et al. 2013, 2014). The elevated blood pressure with tail cuff measurement in programmed rats is consistent with an increase in sympathetic nerve activity (Mizuno et al. 2013, 2014). Rats were euthanized after measurement of blood pressure when they were approximately 3 months of age.

### Serum hormone assays

The following kits were used to measure hormone concentrations as per the manufacturer's instructions: Angiotensin II Enzyme Immunoassay Kit from SPI‐Bio (Montigny le Bretonneux, France), Aldosterone ELISA kit (Enzo Life Sciences, Farmingdale NY) and Renin Activity ELISA (ALPCO, Salem, NH).

### RNA isolation and qPCR

RNA was isolated from rat kidney cortex samples using the TRIzol Reagent method (Invitrogen, Carlsbad, CA). Following DNase treatment of the RNA, the cDNA samples were prepared by the Thermoscript RT‐PCR System (Invitrogen, Carlsbad, CA). TaqMan gene expression assays were used for Real‐Time PCR. Twelve samples from each group were tested for Angiotensinogen or Renin expression and normalized to corresponding 18S ribosomal RNA expression. Each sample was amplified in duplicate on a 96 well plate utilizing a Bio‐Rad CFX Connect Real‐Time System (Bio‐Rad Laboratories, Hercules, CA). The primer/probe sets Angiotensinogen (Rn00593114_m1), Renin (Rn00561847_m1), eukaryotic 18S (Hs99999901_s1) and TaqMan Universal Master Mix II, no UNG (4440040) were all purchased from Thermo Fisher Scientific (Waltham, MA).

### Protein isolation, SDS‐PAGE, and immunoblotting

The kidney was weighed and homogenized in buffer containing 250 mmol/L sucrose and 10 mmol/L triethanolamine, a protease inhibitor cocktail (10 *μ*L/mL) (Sigma St. Louis MO) and phenyl‐methyl‐sulfonyl fluoride (100 *μ*g/mL) (Research Products International, Mount Prospect, Il). After centrifugation at 1000*g* for 15 min, the protein concentration was estimated using the Bradford assay (Bio‐Rad, Hercules, CA).

Twenty micrograms of kidney cortex lysates was denatured at 65°C for 15 min before loading onto polyacrylamide gels. The proteins were separated using SDS‐PAGE and then transferred to a polyvinylidene fluoride membranes which were subsequently blocked with Blotto (5% nonfat dry milk in PBS) for 1 h before incubation with the primary rabbit antibody to rat/mouse angiotensinogen at a 1 *μ*g/mL dilution (Immuno‐Biological Laboratories, Japan) at 4°C overnight. All blots were also probed with an antibody to *β*‐actin at a 1:7500 dilution to ensure equal loading (Sigma, St Louis MO). After primary antibody incubation, the blots were washed and incubated with donkey anti‐rabbit or anti‐mouse secondary antibodies conjugated to horseradish peroxidase at a 1:10,000 dilution (Santa Cruz Biotechnology, Inc., Santa Cruz, CA). Bound antibodies were detected by chemiluminescence and quantitated using densitometry.

### Chemicals

Unless otherwise specified all chemicals were obtained from Sigma Chemical Company (St. Louis, MO).

### Statistics

Data are presented as the mean ± SEM. The data from different groups were compared using analysis of variance with a post hoc Student–Newman–Keuls Test. An unpaired Student's *t*‐test was used when two groups were compared.

## Results

**Figure 1:**
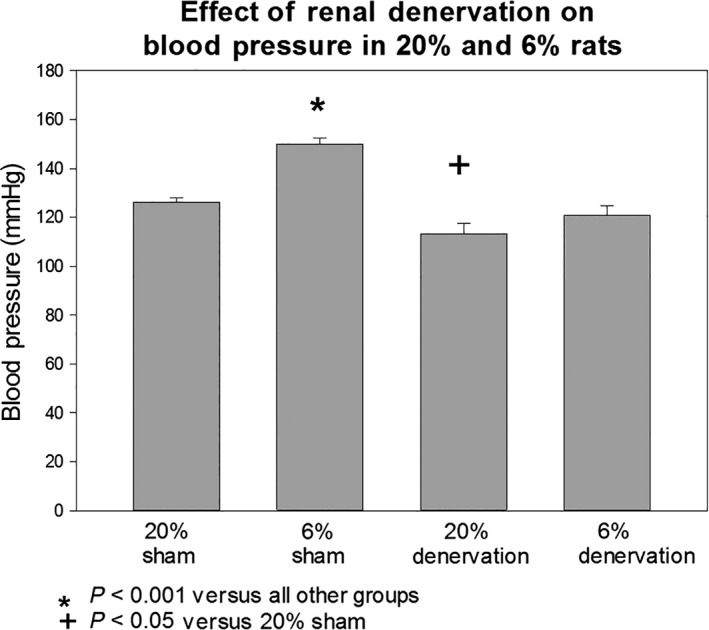
Effect of programming and renal denervation of blood pressure. Blood pressure was measured by tail cuff in adult rats whose mothers were fed either a 20% or a 6% protein diet during the last half of pregnancy. Rats received either a sham operation or renal denervation between 2 and 3 weeks prior to measurement of blood pressure. Blood pressures were measured in a blinded fashion in trained rats. Sham‐operated 6% rats had a higher blood pressure than all other groups (*P* < 0.001). Renal denervation normalized the blood pressure in the 6% group to a level comparable to the 20% sham rats. There was a small but significant decrease in blood pressure in the 20% group with renal denervation compared to the 20% sham group.

Blood pressure was measured in 20% sham‐operated rats, 6% sham‐operated rats, 20% denervated rats and 6% denervated rats. As shown in Figure 1, the blood pressure in the 6% sham‐operated rats was higher than the 20% sham rats. Renal denervation resulted in a reduction in blood pressure in the 6% group to a level comparable to the 20% sham rats. Renal denervation in the 20% rats caused a smaller but significant reduction in blood pressure compared to the 20% sham rats. Thus, renal sympathetic nerves play a significant role in the increase in blood pressure with prenatal programming.

We examined the effect of prenatal programming and renal denervation on urinary protein and albumin excretion, which is shown in Table 1. The 6% sham and 6% denervation rats had higher urinary protein excretion than the 20% sham rats and 20% denervation groups, but this was only statistically significant for protein/creatinine ratio in the 6% denervation group. The urinary albumin excretion rates were greater in the 6% sham than the 20% sham and 20% denervation groups. Renal denervation did not significantly reduce urinary protein or albumin excretion in either the 20% or the 6% groups.

The effect of prenatal programming and renal denervation on creatinine clearance is shown in Table 2. Creatinine clearance was comparable in the 20% sham, 20% denervation, and 6% sham groups. The 6% denervation group had a lower creatinine clearance than the 20% sham and 20% denervation group. The creatinine clearance in the 6% denervation group was lower than the 6% sham only when the two were compared together by a Student's *t‐* test. Table 2 also shows the results of the fractional excretion of sodium, which is the fraction of the filtered sodium which is excreted. The 20% sham and 20% denervation groups were comparable. However, the 6% sham group had a lower fractional excretion of sodium than the other groups. The denervated 6% group had the highest fractional excretion of sodium consistent with renal nerves mediating the increase in sodium reabsorption in programmed rats.

**Table 2:** Effect of prenatal programming and renal denervation on creatinine clearance and fractional excretion of sodium

	Creatinine clearance (ml/min)	Fractional excretion of sodium (%)
20% Sham (*n* = 13)	1.25 ± 0.17	0.50 ± 0.05
6% Sham (*n* = 15)	1.00 ± 0.06	0.20 ± 0.01^c^
20% Denervation (*n* = 12)	1.16 ± 0.14	0.56 ± 0.08
6% Denervation (*n* = 12)	0.74 ± 0.04^a^^,^ b	0.81 ± 0.11^d^

a *P* < 0.05 versus 20% sham and 20% denervation.

b *P* < 0.01 versus 6% sham by Student's t‐test.

c *P* < 0.01 versus other groups.

d *P* < 0.05 versus other groups.

**Figure 2:**
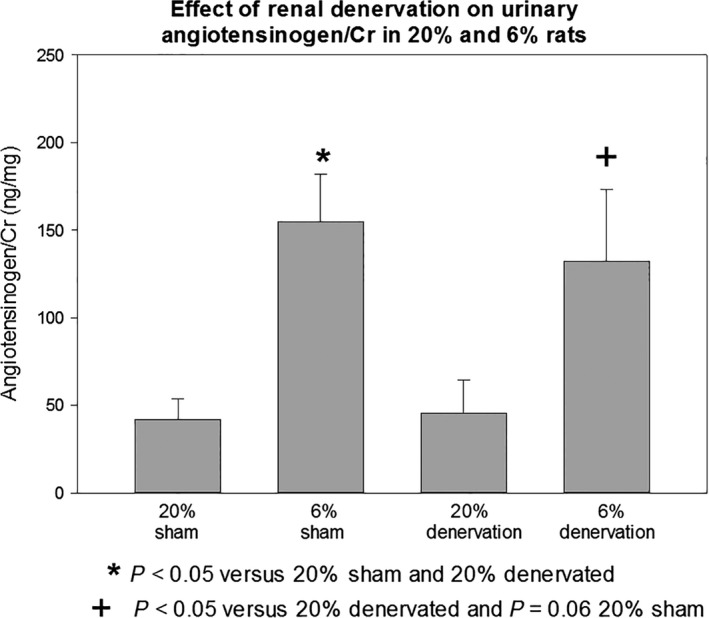
Effect of renal prenatal programming and renal denervation on urinary angiotensinogen/Cr.6% and 20% had a sham operation or renal denervation at 2 months of age. The rats were placed in metabolic cages approximately 1 week after surgery. The rats were provided free access to food and water. After 48 h of acclimation to the metabolic cage, urine was collected for angiotensinogen and creatinine. The 6% sham and 6% denervated rats had higher urinary angiotensinogen/creatinine levels than the respective 20% sham and 20% denervated rats (*P* < 0.05). Denervation did not have a significant effect on urinary angiotensinogen/creatinine levels.

The results of the systemic renin–angiotensin system are shown in Table 3. Plasma renin activity was comparable in the 20% sham and 20% denervation groups. The plasma renin activity was significantly lower in the 6% sham group than the 20% sham and denervation groups but was normalized with renal denervation. The plasma aldosterone level was comparable in the 20% sham, 20% denervation, and the 6% sham groups. The plasma aldosterone level was significantly higher in the 6% denervation groups than the other groups. We examined angiotensinogen and renin mRNA abundance in the four groups which is shown in Table 4. Neither programming nor renal denervation significantly affected angiotensinogen/18s or renin/18s mRNA abundance nor renal cortical angiotensinogen/*β*‐actin protein abundance. Finally, the effect of programming and renal denervation was assessed on urinary angiotensinogen/creatinine, a marker of the intrarenal renin–angiotensin system, which is shown in Figure 2. The 6% sham rats had a higher urinary angiotensinogen/creatinine level than the 20% sham group but there was no significant effect of renal denervation on either the 20% or the 6% groups. Thus, the renal nerves do not have a significant effect on the intrarenal renin–angiotensin system in the model of prenatal programming.

**Table 3:** Effect of prenatal programming and renal denervation systemic renin‐angiotensin system

	Plasma renin activity (ng/mL/h)	Angiotensin II (pg/mL)	Aldosterone (pg/mL)
20% Sham (*n* = 14)	16.9 ± 2.6	79.5 ± 19.0	350 ± 78
6% Sham (*n* = 15)	8.0 ± 1.4^a^	112.4 ± 41.9	537 ± 142
20% Denervation (*n* = 12)	15.8 ± 3.6	97.8 ± 41.9	369 ± 83
6% Denervation (*n* = 13)	19.3 ± 3.0	60.6 ± 12.7	1612 ± 585^a^

a *P* < 0.05 versus other groups.

**Table 4:** Effect of prenatal programming and renal denervation renal angiotensinogen/18s and renin/18s mRNA abundance

	Angiotensinogen/18s mRNA	Renin/18s	Angiotensinogen/*β*‐Actin Protein Abundance
20% Sham	1.04 ± 0.13	1.02 ± 0.17	0.93 ± 0.06
6% Sham	0.75 ± 0.09	1.44 ± 0.26	0.78 ± 0.06
20% Denervation	0.95 ± 0.23	1.50 ± 0.24	0.78 ± 0.08
6% Denervation	1.13 ± 0.21	1.92 ± 0.40	0.71 ± 0.09

## Discussion

Our laboratory and others have previously provided evidence that sympathetic nerves play an important role in mediating the hypertension in prenatal programming (Alexander et al. 2005; Ojeda et al. 2007; Dagan et al. 2008; Mizuno et al. 2013, 2014). We have shown that renal sympathetic nerve activity is augmented to a greater extent in programmed rats compared to control rats in response to the exercise pressor reflex (Mizuno et al. 2013, 2014). Renal denervation normalized the blood pressure in adult offspring that were programmed to develop hypertension as a result of surgically induced uterine perfusion and maternal prenatal administration of dexamethasone (Alexander et al. 2005; Ojeda et al. 2007; Dagan et al. 2008). The finding in this study showing that renal denervation normalized blood pressure in programmed rats whose mothers were fed a low protein diet is in agreement with previous results studying other methods to program rats.

The proximal tubule produces luminal angiotensin II at a concentration 100‐ to 1000‐fold greater than that in blood (Seikaly et al. 1990; Braam et al. 1993). When the glomerular filtrate is obstructed by an oil block or when proximal tubules are perfused in vitro, the addition of an angiotensin II receptor antagonist or a converting enzyme inhibitor into the tubular lumen inhibits proximal tubular transport consistent with the endogenous proximal tubule renin–angiotensin system regulating proximal tubular transport (Quan and Baum 1996; Baum et al. 1997). Our laboratory has demonstrated that the intrarenal renal angiotensin II regulation of proximal tubular transport is modulated by renal nerves (Quan and Baum 2001, 2002). Stimulation of renal sympathetic nerves augments the effect of the intrarenal renin–angiotensin system on proximal tubule transport sodium absorption and renal denervation abrogates the effect of the endogenous renin–angiotensin system on proximal tubular transport (Quan and Baum 2001, 2002).

Whole animal studies have also demonstrated that renal sympathetic nerve activity affects the intrarenal renin–angiotensin system. Urinary angiotensinogen is produced predominantly by the kidney and is a marker of the intrarenal renin–angiotensin system (Kobori et al. 2001, 2002, 2003). Renal angiotensinogen mRNA expression has been shown to be increased by low but not high level of renal nerve stimulation in Wistar rats (Nakamura and Johns 1994, 1995). However, this same group found no effect of renal nerve stimulation on angiotensinogen mRNA expression in spontaneously hypertensive rats (Nakamura and Johns 1995). Others have found that renal nerve stimulation increased urinary angiotensinogen along with a decrease in urine sodium in Wistar rats (Pontes et al. 2015). In this study, we found an increase in urinary angiotensinogen/creatinine ratio in 6% programmed rats which we have shown have an increase in renal sympathetic nerve activity compared to 20% rats (Mizuno et al. 2013, 2014), but renal denervation did not decrease urinary angiotensinogen/creatinine levels. This finding is comparable to the findings above in the spontaneously hypertensive rat where sympathectomy reduced blood pressure in spontaneously hypertensive rats but had no effect on urinary angiotensinogen excretion (Gao et al. 2016). Thus, while renal nerves modulate the intrarenal renin–angiotensin system in control rats, renal denervation does not have a consistent effect to decrease urinary angiotensinogen in the spontaneously hypertensive rat or in programmed rats. The normalization in blood pressure and the increase in urinary sodium excretion in programmed rats with denervation without a change in urinary angiotensinogen/creatinine levels is consistent with renal sympathetic nerves not playing a role to increase the intrarenal renin angiotensin system.

This study examined the effect of renal prenatal programming and renal denervation on the systemic renin–angiotensin system. Using the same model of prenatal programming where the mothers were fed a 6% protein diet during the last half of pregnancy, Vehaskari showed that programmed rats had a lower plasma renin activity (Vehaskari et al. 2001). We also found that plasma renin activity was reduced in the 6% sham rats compared to the 20% sham rats. The renin level in the 6% denervated rats was comparable to the 20% sham and 20% denervated rats. The suppressed plasma renin activity in sham 6% rats is likely due to an increase in extracellular volume mediated by the increase in renal sodium absorption due to an increase in renal nerve activity. This hypothesis was put forth by Vehaskari (Vehaskari et al. 2001). Our results showing a reduced fractional excretion of sodium in the 6% sham compared to the 20% sham which is consistent with an increase in sodium absorption in programmed rats. The increased sodium excretion with renal denervation in the programmed rats, reflected by a higher fractional excretion of sodium, may cause relative volume contraction resulting in the lower creatinine clearance in the 6% denervated group than in 20% groups and 6% sham rats. This could account for the increase in serum aldosterone seen in denervated 6% programmed rats compared to the other groups.

We have shown that prenatal programming with maternal dietary protein deprivation causes a reduction glomerular filtration rate when the rats are approximately 1.5 years of age (Lozano et al. 2015; Mansuri et al. 2017). While programmed rats had an increase in glomerulosclerosis, there was no increase in interstitial fibrosis or increase in renal cortical collagen content (Lozano et al. 2015; Mansuri et al. 2017). In this study, the creatinine clearance was somewhat lower in the 6% sham than the 20% sham, but the difference was not significant. However, the programmed rats had a higher urine albumin and albumin/creatinine ratio than the 20% controls which may reflect an early sign of renal injury in the programmed rats. Unfortunately, urine histology was not determined in this study to determine if there parallel histologic changes.

In conclusion, this study examined the hypothesis that the increase in urinary angiotensinogen, a reflection of the intrarenal renin–angiotensin system, is mediated by renal nerves. We find that renal denervation in programmed rats, but not control rats, has a significant effect on creatinine clearance and fractional sodium excretion. However, there was no effect of renal denervation on urinary angiotensinogen/creatinine suggesting that the intrarenal renin–angiotensin system is dysregulated by prenatal programming by factors other than renal nerves.

## Conflict of Interest

None declared.
